# Tracheostomy in 80 COVID-19 Patients: A Multicenter, Retrospective, Observational Study

**DOI:** 10.3389/fmed.2020.615845

**Published:** 2020-12-17

**Authors:** Yun Tang, Yongran Wu, Fangfang Zhu, Xiaobo Yang, Chaolin Huang, Guo Hou, Wenhao Xu, Ming Hu, Lu Zhang, Aiguo Cheng, Zhengqin Xu, Boyi Liu, Song Hu, Guochao Zhu, Xuepeng Fan, Xijing Zhang, Yadong Yang, Huibin Feng, Lixia Yu, Bing Wang, Zhiqiang Li, Yong Peng, Zubo Shen, Shouzhi Fu, Yaqi Ouyang, Jiqian Xu, Xiaojing Zou, Minghao Fang, Zhui Yu, Bo Hu, You Shang

**Affiliations:** ^1^Department of Critical Care Medicine, Union Hospital, Tongji Medical College, Huazhong University of Science and Technology, Wuhan, China; ^2^Department of Critical Care Medicine, Zhongnan Hospital of Wuhan University, Wuhan, China; ^3^Research Center for Translational Medicine, Wuhan Jinyin-Tan Hospital, Wuhan, China; ^4^Department of Critical Care Medicine, Renmin Hospital, Wuhan University, Wuhan, China; ^5^Department of Critical Care Medicine, Xiaogan Central Hospital, Xiaogan, China; ^6^Department of Critical Care Medicine, Wuhan Pulmonary Hospital, Wuhan, China; ^7^Department of Critical Care Medicine, Xiangyang Central Hospital, Affiliated Hospital of Hubei University of Arts and Science, Xiangyang, China; ^8^Department of Critical Care, The Third People's Hospital of Yichang, Yichang, China; ^9^Department of Critical Care Medicine, Xiangyang No.1 People's Hospital, Affiliated Hospital of Hubei University of Medicine, Xiangyang, China; ^10^Department of Critical Care Medicine, Taihe Hospital Affiliated to Hubei University Medicine, Shiyan, China; ^11^Department of Critical Care Medicine, Fifth Hospital of Wuhan, Wuhan, China; ^12^Department of Critical Care Medicine, The Affiliated Hospital of Jianghan University, Wuhan, China; ^13^Department of Critical Care, Wuhan No.1 Hospital, Wuhan, China; ^14^Intensive Care Unit (ICU) Center of Xijing Hospital, Airforce Medical University, Xi'an, China; ^15^Department of Critical Care Medicine, Huanggang Central Hospital, Huanggang, China; ^16^Department of Intensive Care Unit (ICU), Huangshi Central Hospital, Affiliated Hospital of Hubei Polytechnic University, Edong Healthcare Group, Huangshi, China; ^17^Department of Critical Care Medicine, Jingzhou Central Hospital, The Second Clinical Medical College, Yangtze University, Jingzhou, China; ^18^Department of Critical Care Medicine, No.2 Hospital of Huangshi, Huangshi, China; ^19^Department of Critical Care Medicine, The First People's Hospital of Jingmen, Jingmen, China; ^20^Intensive Care Unit, Xiehe Wuhan Red Cross Hospital, Wuhan, China; ^21^Department of Critical Care Medicine, Ezhou Central Hospital, Ezhou, China; ^22^Department of Intensive Care Unit (ICU)/Emergency, Wuhan Third Hospital, Wuhan, China; ^23^Department of Critical Care Medicine, Tongji Hospital, Tongji Medical College, Huazhong University of Science and Technology, Wuhan, China

**Keywords:** COVID-19, tracheostomy, mechanical ventilation, intensive care unit, critically ill patients

## Abstract

**Background:** The outbreak of coronavirus disease 2019 (COVID-19) has led to a large and increasing number of patients requiring prolonged mechanical ventilation and tracheostomy. The indication and optimal timing of tracheostomy in COVID-19 patients are still unclear, and the outcomes about tracheostomy have not been extensively reported. We aimed to describe the clinical characteristics and outcomes of patients with confirmed severe acute respiratory syndrome coronavirus 2 (SARS-CoV-2) pneumonia who underwent elective tracheostomies.

**Methods:** The multi-center, retrospective, observational study investigated all the COVID-19 patients who underwent elective tracheostomies in intensive care units (ICUs) of 23 hospitals in Hubei province, China, from January 8, 2020 to March 25, 2020. Demographic information, clinical characteristics, treatment, details of the tracheostomy procedure, successful weaning after tracheostomy, and living status were collected and analyzed. Data were compared between early tracheostomy patients (tracheostomy performed within 14 days of intubation) and late tracheostomy patients (tracheostomy performed after 14 days).

**Results:** A total of 80 patients were included. The median duration from endotracheal intubation to tracheostomy was 17.5 [IQR 11.3–27.0] days. Most tracheotomies were performed by ICU physician [62 (77.5%)], and using percutaneous techniques [63 (78.8%)] at the ICU bedside [76 (95.0%)]. The most common complication was tracheostoma bleeding [14 (17.5%)], and major bleeding occurred in 4 (5.0%) patients. At 60 days after intubation, 31 (38.8%) patients experienced successful weaning from ventilator, 17 (21.2%) patients discharged from ICU, and 43 (53.8%) patients had died. Higher 60 day mortality [22 (73.3%) vs. 21 (42.0%)] were identified in patients who underwent early tracheostomy.

**Conclusions:** In patients with SARS-CoV-2 pneumonia, tracheostomies were feasible to conduct by ICU physician at bedside with few major complications. Compared with tracheostomies conducted after 14 days of intubation, tracheostomies within 14 days were associated with an increased mortality rate.

## Introduction

The novel coronavirus disease 2019 (COVID-19), caused by severe acute respiratory syndrome coronavirus 2 (SARS-CoV-2), has resulted in a worldwide pandemic and a large and increasing number of patients who are critically ill and require endotracheal intubation and mechanical ventilation (1–3).

Tracheostomy is a common procedure for critically ill patients who require long-term mechanical ventilation (4). Compared with an orotracheal tube, a shorter tracheostomy tube that bypasses the mouth and pharynx can avoid oropharyngeal and laryngeal lesions, improve patient comfort and reduce sedative drug use (5). In addition, a tracheostomy tube can provide less airway dead space and thus less work of breathing, facilitate weaning from mechanical ventilation, make airway suctioning much easier, and potentially reduce the incidence of ventilator-associated pneumonia (6). In COVID-19 patients with requirements of prolonged ventilation, tracheostomy is one of the important clinical considerations for optimal management (7). However, in the current pandemic, there is significant uncertainty regarding the indication and timing of tracheostomy.

Several recommendations and guidelines have discussed on when to perform a tracheostomy in COVID-19 patients, while the timing is varied across the literature. Recommendations from the UK and North America suggested that tracheostomy should be delayed until at least 14 days from endotracheal intubation to allow prognostic information to become clear and for viral load to sufficiently decline (1, 8–12). In contrast, recommendations from France proposed a more aggressive approach-favoring early tracheostomy so that patients can be weaned off intubation and transferred to a ventilatory weaning unit thus sparing ICU beds for new patients (13). These recommendations were based on expert opinion, and robust ICU outcome data are needed to give high level of evidence. At present, the outcomes about tracheostomy in COVID-19 patients have not been extensively reported.

In this study, we aimed to describe the clinical characteristics of patients with confirmed SARS-CoV-2 pneumonia who underwent elective tracheostomies and to explore the association between the timing of tracheostomy and the outcomes of these patients.

## Materials and Methods

### Study Design and Patients

This multicenter, retrospective, observational study was conducted in Hubei Province, China. Patients treated in intensive care units (ICUs) of 23 hospitals from January 8, 2020 to March 25, 2020 were screened. All patients who were diagnosed with COVID-19 and underwent elective tracheostomies were included. COVID-19 was diagnosed according to the World Health Organization interim guidance (14). The decision of tracheostomy was made by treating clinicians. This study was approved by the Ethics Committee of Union Hospital, and written informed consent was waived.

### Data Collection

Medical records of patients were reviewed, and data were collected by investigators at each ICU by using a standardized case-report form. Sociodemographic and clinical data were collected for all patients, including age, sex, chronic medical histories, vital signs, laboratory tests, acute physiology and chronic health evaluation II (APACHE II) scores and sequential organ failure assessment (SOFA) scores. We also collected details of the tracheostomy procedure, including timing, type (percutaneous or surgical), location, the clinicians performing the procedure, and complications. Whether successful weaning was achieved was also recorded, and successful weaning was defined as no need for mechanical ventilation for more than 48 h at any time after tracheostomy. Treatment was recorded for the duration of hospitalization. The living status at 60 days after intubation was also recorded.

### Statistical Analysis

Normally distributed and non-normally distributed continuous variables are presented as the mean (SD) and median [IQR], respectively. Categorical variables are presented as numbers (%). Early tracheostomy was defined as tracheostomy within 14 days of intubation, and late tracheostomy was defined as tracheostomy after 14 days. The comparison between the two groups was conducted using Student's *t*-test, Mann-Whitney U test or Fisher's exact test when appropriate. The Kaplan-Meier method was used to depict survival curves, and the log-rank test was used to compare the survival rates between the early tracheostomy group and the late tracheostomy group. Cox proportional hazards regression analysis was used to explore the hazard ratio (HR) of variables with a *p* < 0.05 in univariate analysis. No imputation was made for missing data. A 2-tailed *p* < 0.05 was regarded as statistically significant. All statistical analyses were performed using the SPSS software system (vision 20.0, SPSS, Inc., Chicago, IL) and GraphPad Prism 5 software.

## Results

From January 8 to March 25, 2020, a total of 80 patients from 23 hospitals (2 [IQR 1–4] patients per center) in Hubei Province, China, were included in our study. Their mean (SD) age was 63.9 (14.0) years, and 61 (70.1%) were male. The median duration from intubation to tracheostomy was 17.5 [IQR 11.3–27.0] days. Sixty (69.0%) patients had chronic medical illnesses, and the most common illnesses were hypertension (40.0%), coronary heart disease (21.1%), diabetes (17.5%), and cerebrovascular disease (10.0%) (Table 1). Thirty (37.5%) patients received tracheostomies within 14 days after intubation, and their median duration between intubation and tracheostomy was significantly shorter than that of the late tracheostomy group (9.5 [IQR 5.0–13.0] days vs. 24.5 [IQR 18.8–32.0] days, *p* < 0.001). Compared with patients in the early tracheostomy group, the patients in the late tracheostomy group had lower SOFA scores (5 [IQR 4–7] vs. 6 [IQR 4–9], *p* = 0.014) and APACHE II scores (11 [IQR 9–17] vs. 15 [IQR 11–21], *p* = 0.034) at ICU admission and lower APACHE II scores [13 (SD 4) vs. 17 (SD 6), *p* = 0.010] before tracheostomy. Among all 80 patients, lymphocytopenia and hypoalbuminemia at hospital admission and hypoxemia at ICU admission were prominent (Table 2). However, no differences were identified between the two groups.

**Table 1:** Demographic data and vital signs in 80 COVID-19 patients receiving early and late tracheostomies.

	**Total (*n* = 80)**	**Early tracheotomy (*n* = 30)**	**Late tracheotomy (*n* = 50)**	***P*-value**
Male	55 (68.8%)	21 (70.0%)	34 (68.0%)	0.852
Age, years	63.9 (14.0)	66.5 (15.1)	62.3 (13.2)	0.194
Duration from intubation to tracheostomy, days	17.5 [11.3, 27.0]	9.5 [5.0, 13.0]	24.5 [18.8, 32.0]	<0.001
**Chronic medical illness**				
Hypertension	32 (40.0%)	12 (40.0%)	20 (40.0%)	1.000
Coronary heart disease	17 (21.2%)	3 (10.0%)	14 (28.0%)	0.057
Diabetes	14 (17.5%)	6 (20.0%)	8 (16.0%)	0.649
Cerebrovascular disease	8 (10.0%)	4 (13.3%)	4 (8.0%)	0.700
Dementia	5 (6.2%)	3 (10.0%)	2 (4.0%)	0.358
Chronic renal disease	4 (5.0%)	2 (6.7%)	2 (4.0%)	0.628
Chronic hepatic disease	4 (5.0%)	0 (0.0%)	4 (8.0%)	0.291
Cancer	3 (3.8%)	2 (6.7%)	1 (2.0%)	0.553
COPD	2 (2.5%)	0 (0.0%)	2 (4.0%)	0.525
**At hospital admission**				
Temperature, °C	36.7 [36.3, 37.2]	36.8 [36.4, 37.0]	36.5 [36.3, 37.3]	0.811
Heart rate, beats per minute	96 (18)	97 (20)	96 (18)	0.800
Respiratory rate	22 [19, 30]	22 [19, 30]	23 [19, 30]	0.731
Systolic blood pressure, mm Hg	133 [123,146]	132 [120, 146]	135 [123, 146]	0.827
Diastolic blood pressure, mm Hg	78 [70, 88]	77 [70, 90]	78 [67, 85]	0.365
**At ICU admission**				
Temperature, °C	36.8 [36.5, 37.2]	36.8 [36.5, 37.5]	36.7 [36.4, 37.2]	0.324
Heart rate, beats per minute	99 (20)	102 (19)	98 (20)	0.334
Respiratory rate	25 [20, 32]	27 [20, 32]	25 [20, 32]	0.988
Systolic blood pressure, mm Hg	133 [121, 148]	136 [114, 155]	133 [123, 146]	0.769
Diastolic blood pressure, mm Hg	75 [65, 87]	76.5 [65, 88]	75 [65, 83]	0.754
SOFA score	5 [4, 7]	6 [4, 9]	5 [4, 7]	0.014
APACHE II score	12 [9, 18]	15 [11, 21]	11 [9, 17]	0.034
**On the day before tracheostomy**				
Temperature, °C	37 [36.6, 37.7]	36.8 [36.4, 37.7]	37 [36.7, 37.7]	0.260
Heart rate, beats per min	96 (20)	93 (26)	97 (16)	0.212
Respiratory rate	21 [19, 25]	22 [19, 25]	21 [20, 23]	0.595
Systolic blood pressure, mm Hg	129 (18)	129 (21)	129 (16)	0.949
Diastolic blood pressure, mm Hg	70 [61, 78]	69 [60, 78]	71 [62, 78]	0.835
SOFA score	7 [5, 10]	8 [6, 10]	7 [5, 9]	0.371
APACHE II score	15 (5)	17 (6)	13 (4)	0.010

*Data were presented as mean (standard deviation), median [interquartile range] or count (%)*.

*COVID-19, coronavirus disease 2019; ICU, intensive care unit; COPD, chronic obstructive pulmonary disease; SOFA, sequential organ failure assessment; APACHE, acute physiology and chronic health evaluation*.

**Table 2:** Laboratory tests in 80 COVID-19 patients receiving early and late tracheostomies.

	**Normal range**	**Total (*n* = 80)**	**Early tracheotomy (*n* = 30)**	**Late tracheotomy (*n* = 50)**	***P*-value**
**At hospital admission**					
White-cell count, × 10^9^ /L	3.5–9.5	8.7 [6.2, 12.6]	9.5 [6.8, 11.4]	8.4 [5.7, 14.6]	0.518
Hemoglobin, g/L	130–175	124.0 [104.5, 136.0]	125.0 [103.5, 138.5]	124.0 [103.8, 136.0]	0.760
Platelet count, × 10^9^ /L	125–350	154.5 [111.8, 204.8]	152.0 [101.5, 233.0]	157.0 [118.0, 200.5]	0.971
Neutrophil count, × 10^9^ /L	1.8–6.3	7.8 [4.7, 11.9]	8.4 [6.1, 10.0]	7.4 [4.4, 14.3]	0.580
Lymphocyte count, × 10^9^ /L	1.1–3.2	0.64 [0.42, 0.96]	0.60 [0.40, 0.93]	0.66 [0.43, 1.00]	0.435
Total bilirubin, μmol/L	0–26	15.3 [10.3, 22.4]	16.1 [10.3, 24.1]	15.0 [10.3, 20.6]	0.388
ALT, U/L	9–50	32.0 [21.0, 57.0]	29.0 [20.5, 60.0]	32.0 [22.0, 55.0]	0.683
AST, U/L	15–40	37.5 [24.0, 58.3]	38.0 [23.0, 63.5]	37.0 [27.7, 56.5]	0.852
Albumin concentration, g/L	40–55	32.0 (5.7)	31.5 (6.1)	32.2 (5.5)	0.588
Serum creatinine, μmol/L	57–111	72.1 [54.9, 92.0]	78.8 [55.5, 128.6]	70.7 [54.8, 84.4]	0.174
Blood urea nitrogen, mmol/L	3.6–9.5	7.1 [5.1, 9.9]	8.3 [5.3, 11.5]	6.7 [4.9, 9.4]	0.200
C-reactive protein, mg/L	0–5	73.2 [16.8, 115.5]	57.2 [15.0, 134.0]	76.1 [19.4, 111.5]	0.631
Procalcitonin, ng/mL	<0.5	0.17 [0.08, 0.43]	0.29 [0.10, 0.78]	0.14 [0.08, 0.40]	0.075
**At ICU admission**					
PH	7.35–7.45	7.42 [7.36, 7.47]	7.42 [7.39, 7.47]	7.41 [7.34, 7.48]	0.737
PaO_2_, mm Hg	83–108	68.4 [54.0, 97.0]	68.9 [57.6, 90.8]	66.0 [53.0, 108.0]	0.680
PaCO_2_, mm Hg	35–48	37.0 [33.0, 48.3]	36.4 [32.2, 46.2]	41.0 [34.0, 49.0]	0.250
Ratio of PaO_2_ to FiO_2_, mm Hg	400–500	112.0 [72.7, 178.7]	108.5 [63.1, 178.8]	114.0 [75.0, 178.7]	0.552
**On the day before tracheostomy**					
PH	7.35–7.45	7.41(0.07)	7.41(0.07)	7.41(0.07)	0.671
PaO_2_, mm Hg	83–108	88.5 [72.9, 113.5]	82.3 [67.0, 94.8]	91.5 [77.8, 131.3]	0.052
PaCO_2_, mm Hg	35–48	49.2 (12.9)	51.7 (15.1)	47.8 (11.5)	0.226
Ratio of PaO_2_ to FiO_2_, mm Hg	400–500	183.0 [126.0, 268.3]	147.6 [93.0, 253.8]	214.5 [146.3, 279.8]	0.061

*Data were presented as mean (standard deviation) or median [interquartile range]*.

*COVID-19, coronavirus disease 2019; ICU, intensive care unit; ALT, alanine aminotransferase; AST, aspartate aminotransferase; PaO_2_, partial pressure of oxygen; PaCO_2_, partial pressure of carbon dioxide; FiO_2_, fraction of inspired oxygen*.

Most tracheotomies were performed by ICU physicians [62 (77.5%)] and using percutaneous techniques [63 (78.8%)] at the ICU bedside [76 (95.0%)]. Powered air-purifying respirators (PAPRs) were used by operating teams in 68 (85.0%) tracheostomies (Table 3). Furthermore, neuromuscular blocking drugs were applied in 46 (57.5%) patients, which may help avoid coughing-induced viral aerosolization. The most common complication was tracheostoma bleeding, which occurred in 14 (17.5%) patients. Major bleeding occurred in 4 (5.0%) patients, who received transfusion of red blood cells. Other complications included subcutaneous emphysema (2.5%), tracheostoma infection (1.2%), and mediastinal emphysema (1.2%) (Table 3). No differences were identified between the early and late tracheostomy groups in terms of complications. For treatments, no differences were identified between the two groups, except extracorporeal membrane oxygenation (ECMO). Compared with early tracheostomy patients, more patients who underwent late tracheostomy received ECMO [19 (8.0%) vs. 2 (6.7%), *p* = 0.002] (Table 4).

**Table 3:** Details of the Tracheostomies in 80 COVID-19 patients.

	**Total (*n* = 80)**	**Early tracheotomy (*n* = 30)**	**Late tracheotomy (*n* = 50)**	***P*-value**
Type of procedure				0.057
Surgical	17 (21.2%)	3 (10.0%)	14 (28.0%)	
Percutaneous	63 (78.8%)	27 (90.0%)	36 (72.0%)	
Location				0.291
Operating room	4 (5.0%)	0 (0.0%)	4 (8.0%)	
Bedside	76 (95.0%)	30 (100.0%)	46 (92.0%)	
Clinicians performing tracheostomy				0.028
ICU physicians only	62 (77.5%)	28 (93.3%)	34 (68.0%)	
Otolaryngologists only	10 (12.5%)	1 (3.3%)	9 (18.0%)	
Both	8 (10.0%)	1 (3.3%)	7 (14.0%)	
PAPRs	68 (85.0%)	24 (80.0%)	44 (88.0%)	0.518
Neuromuscular blocking drugs	46 (57.5%)	12 (40.0%)	34 (68.0%)	0.014
Complications	18 (22.5%)	5 (16.7%)	13 (26.0%)	0.333
Tracheostoma bleeding	14 (17.5%)	4 (13.3%)	10 (20.0%)	
Subcutaneous emphysema	2 (2.5%)	1 (3.3%)	1 (2.0%)	
Tracheostoma infection	1 (1.2%)	0 (0.0%)	1 (2.0%)	
Mediastinal emphysema	1 (1.2%)	0 (0.0%)	1 (2.0%)	

*Data were presented as count (%)*.

*COVID-19, coronavirus disease 2019; ICU, intensive care unit; PAPRs, powered air-purifying respirator*.

**Table 4:** Treatments in 80 COVID-19 patients receiving early and late tracheostomy.

	**Total (*n* = 80)**	**Early tracheotomy (*n* = 30)**	**Late tracheotomy (*n* = 50)**	***P*-value**
Prone position ventilation	45 (56.2%)	15 (50.0%)	30 (60.0%)	0.383
ECMO	21 (26.2%)	2 (6.7%)	19 (38.0%)	0.002
Renal replacement therapy	37 (46.2%)	14 (46.7%)	23 (46.0%)	0.954
Vasoconstrictive agents	71 (88.8%)	26 (86.7%)	45 (90.0%)	0.927
Antiviral agents	62 (77.5%)	23 (76.7%)	39 (78.0%)	0.890
Antibacterial agents	87 (100.0%)	30 (100.0%)	50 (100.0%)	1.000
Antifungal agents	61 (76.2%)	21 (70.0%)	40 (80.0%)	0.309
Glucocorticoids	53 (66.2%)	20 (66.7%)	33 (66.0%)	0.951

*Data were presented as count (%)*.

*COVID-19, coronavirus disease 2019; ECMO, extracorporeal membrane oxygenation*.

**Figure 1:**
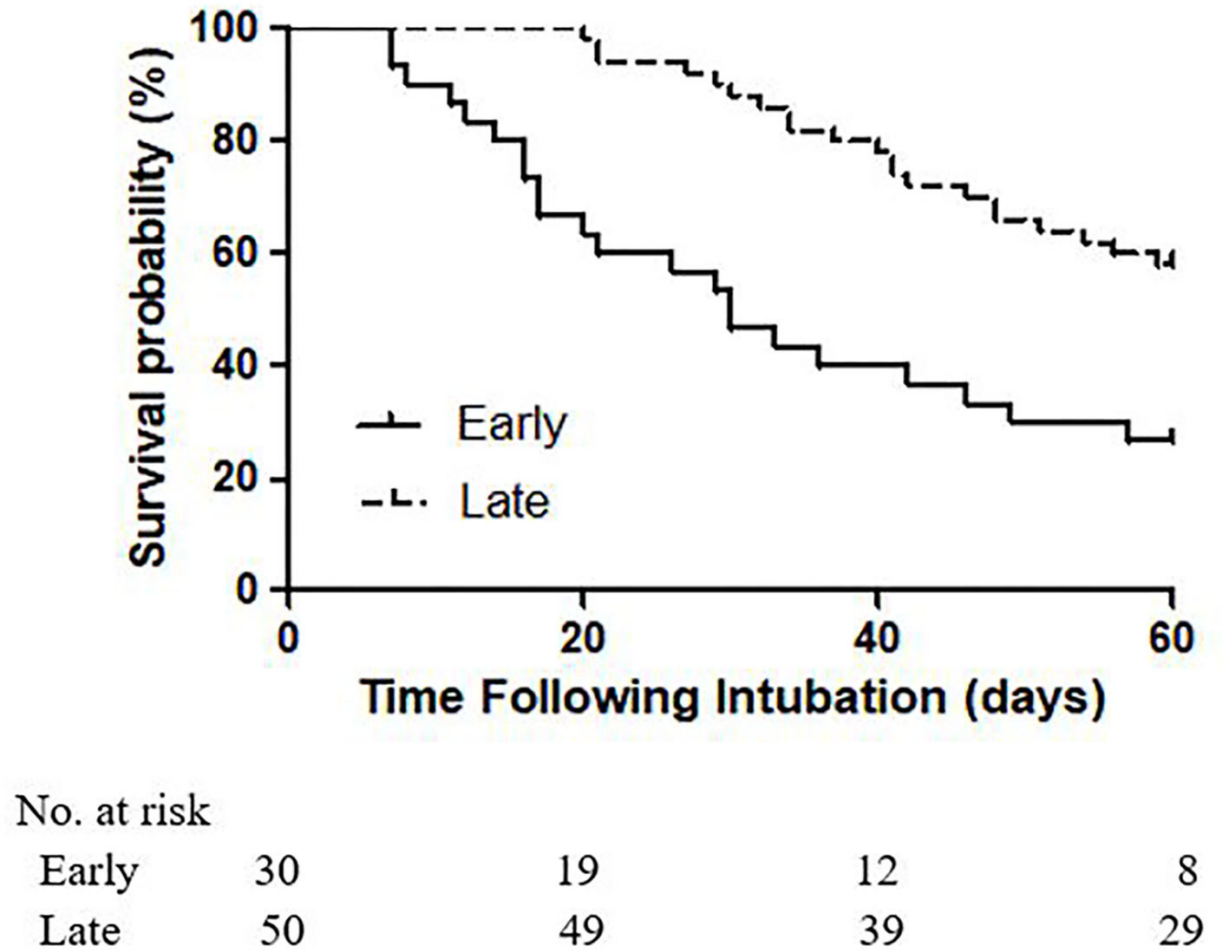
Kaplan-Meier analysis of survival in patients receiving early and late tracheostomies for 60 days (*p*_log−ranktest_ = 0.0003).

In the 80 COVID-19 patients who underwent elective tracheostomies, 43 (53.8%) patients had died at 60 days. Higher 60 day mortality [22 (73.3%) vs. 21 (42.0%), *p* = 0.007] was identified in patients who underwent early tracheostomy (Figure 1). At 60 days after intubation, 31 (38.8%) patients experienced successful weaning from the ventilator, and 17 (21.2%) patients were discharged from the ICU. Because collinearity existed between the SOFA and APACHE II scores at ICU admission, only the SOFA score was incorporated into the Cox proportional hazards regression analysis. After adjusting for SOFA [HR 1.00 (95% CI, 0.91–1.11)] and ECMO [HR 1.06 (95% CI, 0.49–2.28)], late tracheostomy was identified with a decreased risk of death [HR 0.34 (95% CI, 0.17–0.70)] (Table 5).

**Table 5:** Cox proportional hazards regression analysis in 80 COVID-19 patients receiving tracheostomy.

**Characteristics**	**Hazard ratio and 95% confidence interval**	***p*-value**
Late tracheostomy	0.34 (0.17–0.70)	0.003
SOFA	1.00 (0.91–1.11)	0.958
ECMO	1.06 (0.49–2.28)	0.881

*COVID-19, coronavirus disease 2019; SOFA, sequential organ failure assessment; ECMO, extracorporeal membrane oxygenation*.

## Discussion

As the number of patients infected by SARS-CoV-2 around the world is increasing, the demand for endotracheal intubation and invasive mechanical ventilatory support secondary to acute respiratory failure is increasing accordingly (15, 16). In our study, most procedures were performed by ICU physicians using percutaneous techniques at bedside, which avoided the unnecessary transport of ventilated patients and repeated connection and disconnection of ventilatory circuits during transfer. Regarding the type of tracheostomy performance, one of the concerns is complications of bleeding and stomal infections. Long et al. (17) compared percutaneous with surgical tracheotomy in patients with COVID-19, and they found there were no significant differences in complication rates between the two methods. Another concern is the potential risk of viral transmission. Some argued against percutaneous tracheostomy performed in COVID-19 patients because it usually involves opening the ventilator circuit more frequently than surgical tracheostomy, and serial dilations during the procedure may put surgeons in face of the airway from the beginning (18). However, there is currently no evidence across the literature to advise which approach is less aerosol generating (19).

Tracheotomy for patients with COVID-19 is considered a highly-risk procedure, and appropriate personal protective equipment (PPE) is critical to reduce infection rates among health care workers (20). In our study, standard PPE was systematically used in all of the procedures, including N95 mask, gowns, caps, boots, double gloves and face shield/eye protection. Additionally, the PAPR, which was advised by several recommendations (1, 12, 21), was used in more than half of the procedures in our study. Other principles, including limiting the number of personnel present, ensuring complete paralysis, adequate sedation, and minimizing suction during the procedure, also help to improve protection for health care workers from SARS-CoV-2 (22).

Indications for tracheostomy in patients with COVID-19 remain unclear. Mattioli et al. suggested that tracheostomy has the potential to facilitate ventilator weaning and promote early discharge of COVID-19 patients from ICU to lower intensity care wards and thus free up resources (23). However, Shiba et al. argued that tracheostomy does not provide any benefit on the outcome in patients with COVID-19 due to rapid evolution of the disease, and they did not believe that tracheostomy had widespread indication (24). Above all, before consideration of tracheostomy, ICU physicians and surgery teams should fully assess the prognosis and associated benefit from the procedure. Tracheostomy is preferably be offered to patients with an expectation of recovery or a long-term need of an artificial airway.

Timing for elective tracheostomy performance is always controversial. Outside the context of the COVID-19 pandemic, a systematic review suggested that early tracheostomy (within 7 days) was associated with a reduced duration of mechanical ventilation, less mortality rate and shorter length of ICU stay (25). Furthermore, A Cochrane review found lower mortality rates and a higher probability of discharge from the ICU at day 28 among patients with early tracheotomy (26). In contrast, meta-analyses published by Griffiths et al. (27) and Siempos et al. (28) suggested that early tracheostomy is not associated with lower mortality than late tracheostomy. Moreover, a TracMan randomized trial (29), comparing 455 patients undergoing early tracheostomy (within 4 days) and 454 patients undergoing late tracheostomy (after 10 days), found that there were no differences in 30 day mortality and 1 and 2 year survival or length of ICU stay between them. During the pandemic of COVID-19, the focus has changed dramatically. Tracheostomy is an aerosol generating procedure which theoretically increases the risk of viral transmission, and the viral load may be high in the early course of the disease (7, 21). The timing should balance the benefits of tracheostomy for mechanically ventilated patients and the risk of viral transmission to the team involved in the procedure. Both the US and Canadian recommendations strongly advised that test for COVID-19 should be negative before performing an elective tracheostomy (8, 30).

Our study suggested that, compared with tracheostomies conducted after 14 days of intubation, tracheostomies within 14 days were associated with an increased mortality rate. Univariate analysis showed that patients who underwent early tracheostomies had higher SOFA scores and APACHE II scores, and less of these patients received ECMO. However, after adjusting SOFA and ECMO, the timing of tracheostomy was the only variable significantly associated with mortality. A prospective cohort study assessed 50 patients with confirmed COVID-19 reported that early tracheotomy (≤ 10 days) was associated with shorter mechanical ventilation duration and hospital stay, and no differences were found in mortality rate (31). The overall mortality in our study was as high as 53.8%, which was consistent with other studies reported > 50% mortality rate for patients who are placed on the ventilator (2, 32, 33). Given the high mortality rate, lack of proven benefit, and concern for viral exposure, it is reasonable to consider tracheostomy no sooner than 14 days of endotracheal intubation, and preferably at least tests of specimens from the respiratory tract for SARS-CoV-2 RNA are negative.

This study has several limitations. First, the sample size of our study was relatively small, which might cause bias and limit the reliability or generalizability of our results. Second, some patients were still hospitalized at the end of this study, so some clinical outcomes, such as length of ICU stay and hospital stay, were unavailable at the time of analysis. Third, due to its retrospective design, the lack of randomization for patients who underwent early and late tracheostomy may increase the possibility of confounding in the subsequent comparison. Forth, results of SARS-CoV-2 tests from clinicians involved in tracheostomies were not available. Even if they were test positive for SARS-CoV-2-RNA, we were unable to ascertain whether the clinicians contracted it during the procedures. In future research, rigorous prospective randomized trials with large samples are needed to elucidate any potential benefit from tracheostomy in COVID-19 patients and determine the optimal timing of this procedure.

## Conclusion

In patients with severe SARS-CoV-2 pneumonia, tracheostomies were feasible to conduct by ICU physicians at bedside with few major complications. Compared with tracheostomies conducted after 14 days of intubation, tracheostomies within 14 days were associated with an increased mortality rate. Despite the results, further research and data from other institutions are warranted to more accurately verify these findings.

## Data Availability Statement

The original contributions presented in the study are included in the article/Supplementary Materials, further inquiries can be directed to the corresponding author/s.

## Ethics Statement

The studies involving human participants were reviewed and approved by the Ethics Committee of Union Hospital. Written informed consent for participation was not required for this study in accordance with the national legislation and the institutional requirements.

## Author Contributions

YT, YW, FZ, XY, CH, GH, WX, MH, LZ, AC, ZX, BL, SH, GZ, XF, XZh, YY, HF, LY, BW, ZL, YP, ZS, SF, YO, JX, and XZo collected the epidemiological and clinical data. YT, YW, and FZ summarized all the data. YT, YW, FZ, XY, CH, and GH drafted the manuscript. MF, ZY, BH, and YS revised the final manuscript. All authors read and approved the final manuscript.

## Conflict of Interest

The authors declare that the research was conducted in the absence of any commercial or financial relationships that could be construed as a potential conflict of interest.

## Abbreviations

COVID-19: Coronavirus disease 2019
SARS-CoV-2: severe acute respiratory syndrome coronavirus 2
ICU: Intensive care unit
COPD: chronic obstructive pulmonary disease
SOFA: sequential organ failure assessment
APACHE: acute physiology and chronic health evaluation
ECMO: extracorporeal membrane oxygenation
PPE: Personal Protective Equipment
PAPRs: Powered air-purifying respirators.
